# Molecular imaging of potential bone metastasis from differentiated thyroid cancer: a case report

**DOI:** 10.1186/1752-1947-5-522

**Published:** 2011-10-23

**Authors:** Nora Sandu, Gabriele Pöpperl, Marie-Elisabeth Toubert, Belachew Arasho, Toma Spiriev, Mikael Orabi, Bernhard J Schaller

**Affiliations:** 1 Department of Neurological Surgery, Lariboisiere Hospital, Universities of Paris, Paris, France; 2 Department of Neurological Surgery, University of Lausanne, Lausanne, Switzerland; 3 Department of Nuclear Medicine, Hospital of Stuttgart, Stuttgart, Germany; 4 Department of Nuclear Medicine, Hospital of St. Louis, University of Paris, Paris, France; 5 Department of Neurology, University of Addis Ababa, Addis Ababa, Ethiopia

## Abstract

**Introduction:**

Molecular imaging of the spine is a rarely used diagnostic method for which only a few case reports exist in the literature. Here, to the best of our knowledge we present the first case of a combination of molecular imaging by single photon emission computer tomography and positron emission tomography used in post-operative spinal diagnostic assessment.

**Case presentation:**

We present the case of a 50-year-old Caucasian woman experiencing progressive spinal cord compression caused by a vertebral metastasis of a less well differentiated thyroid cancer. Following tumor resection and vertebral stabilization, total thyroidectomy was performed revealing follicular thyroid carcinoma pT2 pNxM1 (lung, bone). During follow-up our patient underwent five radioiodine therapy procedures (5.3 to 5.7 GBq each) over a two-year period. Post-therapeutic I-131 scans showed decreasing uptake in multiple Pulmonary metastases. However, following an initial decrease, stimulated thyroglobulin remained at pathologically increased levels, indicating further neoplastic activity. F18 Fludeoxyglucose positron emission tomography, which was performed in parallel, showed remaining hypermetabolism in the lungs but no hypermetabolism of the spinal lesions correlating with the stable neurological examinations. While on single photon emission computer tomography images Pulmonary hyperfixation of I-131 disappeared (most likely indicating dedifferentiation), there was persistent spinal hyperfixation at the operated level and even higher fixation at the spinal process of L3. Based on the negative results of the spinal F18 fludeoxyglucose positron emission tomography, a decision was made not to operate again on the spine since our patient was completely asymptomatic and the neurological risk seemed to be too high. During further follow-up our patient remained neurologically stable.

**Conclusions:**

Molecular imaging by F18 fludeoxyglucose positron emission tomography helps to exclude metabolically active spinal metastases and to spare further risky surgery.

## Introduction

Fluorine-18 fludeoxyglucose (FDG) positron emission tomography (PET) is a well established diagnostic modality for standard oncological staging, restaging, and treatment monitoring evaluations, and has a major impact on patient management [1-3]. A key issue that is less well studied is the performance of FDG-PET in accurately depicting bone metastases that would potentially have a large effect on patient treatment [2,4]. Metastases to the spine represent a common problem in large oncology centers and usually present a problem in radiological diagnosis. The role of PET is still being assessed in this context.

However, molecular imaging (MI) with FDG-PET seems a good additional state-of-the-art method to demonstrate the viability of previously treated spinal tumor metastasis or to differentiate malignant from benign lesions in the spine [2,4]. Additionally, PET may help to find the sites of the most metabolic active lesions for biopsy [2,5]. In thyroid cancer, PET MI is useful in patients with metastatic poorly differentiated tumors with high thyroglobulin (Tg) levels and negative ^131^I whole-body scan results [6,7].

Similar to the situation with other tumor types, it is currently unclear whether FDG-PET is adequate in the detection of bone metastasis of thyroid cancer. We describe one of the very few reported clinical cases with vertebral metastases of a less well differentiated follicular thyroid carcinoma followed by FDG-PET and I-131 single photon emission computer tomography (SPECT). The unique feature of this case is that the follow-up was performed by FDG-PET and SPECT, and we can therefore compare the results of these two MI modalities. MI by FDG-PET helped to exclude a metabolically active spinal metastasis.

## Case report

We present the case of a 50-year-old Caucasian woman with a vertebral metastasis of a less well differentiated thyroid cancer, who was followed over a three-year period clinically and by spinal FDG-PET and I-131 SPECT imaging after initial surgery. Table 1 chronologically summarizes the treatment modalities, corresponding laboratory test values (thryotropin (TSH) and Tg level) and MI results (FDG-PET and I-131 SPECT) for different time points during follow-up.

**Table 1:** Treatment modalities, corresponding laboratory values (TSH and Tg level) and MI results (FDG-PET and I-131 SPECT) for different time points during follow-up

Parameter	Date (MM/YY format) and treatment								
	
	08/06	10/06	11/06	05/07	11/07	06/08	11/08	10/09	05/10
Treatment	Cementoplasty and posterior arthrodesis	Total thyroidectomy	First RIT (GBq level unknown)	Second RIT (GBq level unknown)	Third RIT (5.4 GBq)	Fourth RIT (5.5 GBq)	Fifth RIT (5.8 GBq)	Wait and see	Wait and see

TSH (mIU/L)	NA	NA	NA	NA	103	119	130	NA	< 0.02

Thyroglobulin (μg/L)	NA	NA	2357	805	891	1035	939	NA	606

I-131 SPECT	NA	NA	Positive uptake: thyroid bed, multiple foci in the lungs, osseous lesions T11, L3, os ilium, left femur	No uptake in the thyroid bed, decreasing uptake in the lungs, stable uptake in the osseous lesions	No uptake in the thyroid bed, os ilium, left femur decreasing uptake in the lungs, stable uptake in the spine lesions T11/L3	No uptake in the thyroid bed, os ilium, left femur decreasing uptake in the lungs, stable uptake in the spine lesions T11/L3	No uptake in the thyroid bed, os ilium, left femur decreasing uptake in the lungs, stable uptake in the spine lesions T11/L3	NA	NA

FDG-PET	NA	NA	NA	NA	NA	NA	Slight uptake in Pulmonary metastases, no uptake in spinal lesions T11/L3	Slight uptake in Pulmonary metastases, no uptake in spinal lesions T11/L3	NA

FDG = fludeoxyglucose; NA = not available; PET = positron emission tomography; RIT = radioiodine treatment; Tg = thyroglobulin; TSH = thryotropin; SPECT = single photon emission computer tomography.

Our patient presented to our facility with progressive spinal cord compression. An MRI scan revealed a vertebral metastasis at the T11 level with intraspinal extension compressing the spinal cord. Our patient was operated on via a bilateral posterolateral approach, allowing for tumor resection and stabilization of her vertebral column by Cementoplasty and a posterior arthrodesis. A histopathological examination concluded 'metastasis of a less well differentiated thyroid carcinoma', which was confirmed after total thyroidectomy (follicular thyroid carcinoma pT2 pNx). Following her first radioiodine therapy a post-therapeutic scan revealed multiple lung metastases and further bone metastases at the L3 level, os ilium and left femur; therefore the tumor was staged as M1 (lung, bone). During follow-up our patient received five radioiodine therapies (5.3 to 5.7 GBq each) in total over a two-year period.

**Figure 1:**
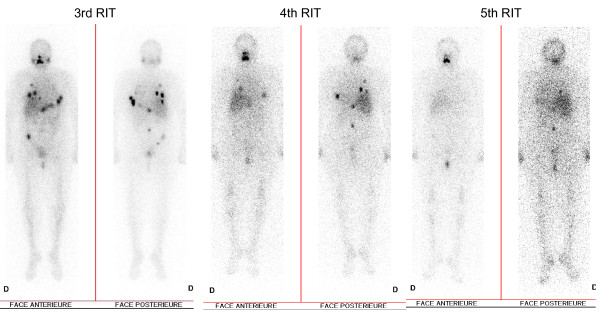
**Planar I-131 whole-body scintigraphies after our patient's third, fourth and fifth radioiodine treatments, demonstrating decreasing uptake in the pulmonary metastases in the right and left lung parenchyma but stable uptake in the spinal lesions of Th11 and L3**.

**Figure 2:**
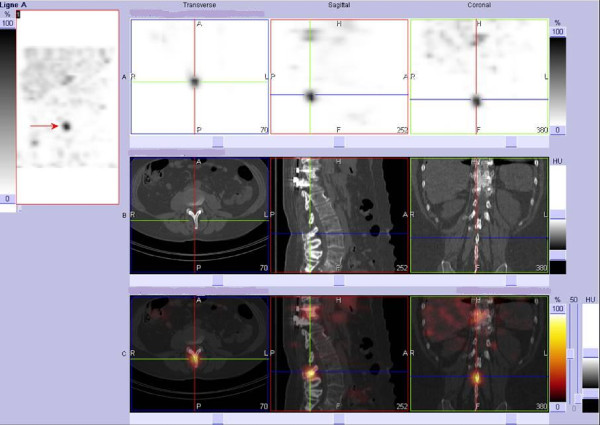
**Single photon emission computer tomography (SPECT) I-131-CT demonstrating a persistent hyperfixation at the operated level and even higher fixation at the spinal process of L3**.

**Figure 3:**
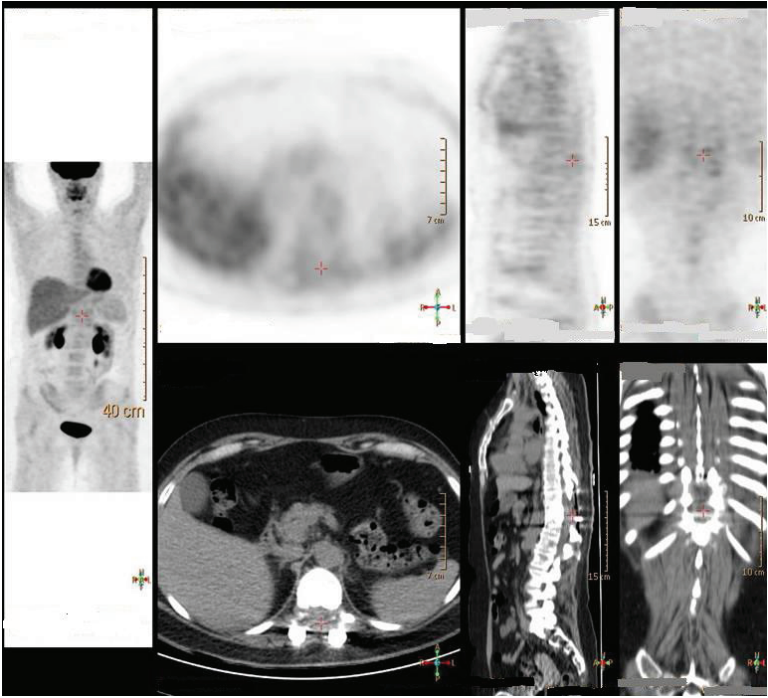
**18F-fludeoxyglucose positron emission tomography/computed tomography (FDG-PET-CT) demonstrating hypometabolism at the spinal level correlating with the stable neurological examination**.

During the follow-up period our patient was regularly monitored clinically and by means of a tumor marker (thyroglobulin), PET-CT ([F-18]-FDG) and post-therapeutic SPECT (I-131). Clinically and neurologically our patient was stable over three years of follow-up. Post-therapeutic radioiodine scans showed decreasing uptake in most Pulmonary lesions but remaining uptake in spine lesions (Figure 1). Her stimulated thyroglobulin blood levels dropped from 2356 μg/L at baseline to 939 μg/L following the last radioiodine treatment. However, even after finishing five radioiodine cycles Tg remained on a pathologically increased level, indicating some neoplastic activity. FDG-PET imaging showed slight but remaining hypermetabolism in the lungs whereas in SPECT imaging Pulmonary hyperfixation of I-131 disappeared, most likely indicating dedifferentiation. On the spinal level, SPECT images showed persistent hyperfixation at the operated level (T11) and even higher fixation at the spinal process of L3 (Figure 2) suggestive for remaining, more differentiated metastases. FDG-PET, however, showed no hypermetabolism, which correlated with the stable neurological examination results (Figure 3).

After thorough interdisciplinary discussion, despite the remaining I-131 uptake it was decided not to operate again on our patient's spine as she was completely asymptomatic; conventional imaging also remained stable and the neurological risk seemed to be too high for the thoracic level. During further follow-up our patient remained neurologically stable.

## Discussion

Detection of spinal metastasis by MI is a relatively new, but clinically important technique. Cases such as our patient's, where the different MI modalities can be directly compared, are important to gain more experience in the different modalities for spinal MI and to perhaps find special indications for the one or the other method. In addition, our case report underlines the usefulness of FDG-PET in assessing the metabolic activity of bone metastasis of less well differentiated thyroid cancer.

In our case report, in which different MI techniques were used for the detection of distant metastases from thyroid cancer, we were able to demonstrate different behavior of the pulmonary and osseous lesions. While the pulmonary nodes presented with decreased radioiodine uptake but increased FDG uptake indicating de-differentiation, the spinal lesions showed stable radioiodine uptake without FDG uptake, most probably indicating stable disease. Subsequently, integrated I-131 SPECT/CT was found to have an additional value compared to planar scintigraphy in patients with thyroid cancer for correct characterization of equivocal tracer uptake seen on planar imaging, as well as for precise localization of malignant lesions in the skeleton [8,9]. In our patient's case these combined MI findings justified not operating again on her spine; this turned out to be the right decision, since our patient remained neurologically stable over further follow-up.

The FDG-PET examinations were performed under stimulated TSH conditions to increase the diagnostic sensitivity. It is known that TSH stimulates thyrocyte metabolism, glucose transport and glycolysis. Since FDG is a glucose analog, several studies have shown that recombinant human TSH (rhTSH) stimulation improves the detection of occult thyroid metastases with FDG-PET, compared with scans performed on TSH suppression [10]. Beyond I-131 targeting the OPG/RANK/RANKL axis may offer a novel therapeutic approach for malignant osteolytic pathologies [11], but currently there are no such studies specifically for thyroid cancer bone metastases.

## Conclusions

The presence of bone metastases alters the prognosis of patients with differentiated thyroid carcinoma. Our case report underlines the fact that FDG-PET can have an important impact on management in patients with thyroid cancer.

## Consent

Written informed consent was obtained from the patient for publication of this case report and any accompanying images. A copy of the written consent is available for review by the Editor-in-Chief of this journal.

## Competing interests

The authors declare that they have no competing interests.

## Authors' contributions

NS, GP, MO and BS analyzed and interpreted the data from our patient regarding the neurosurgical disease and the molecular. MET performed the histological examination of the kidney, and together with NS, GP, MO, BA, TS and BS was a major contributor to writing the manuscript. All authors read and approved the final manuscript.

## References

[B1] Hillner BE Siegel BA Liu D Shields AF Gareen IF Hanna L Stine SH Coleman RE Impact of positron emission tomography/computed tomography and positron emission tomography (PET) alone on expected management of patients with cancer: initial results from the National Oncologic PET Registry J Clin Oncol 2008 26 2155 2161 10.1200/JCO.2007.14.5631 18362365

[B2] Sandu N Pöpperl G Toubert ME Spiriev T Arasho B Orabi M Schaller BJ Current molecular imaging of spinal tumors in clinical practice Mol Med 2011 17 308 316 21210073 10.2119/molmed.2010.00218 PMC3060992

[B3] Schaller B Usefulness of positron emission tomography in diagnosis and treatment follow-up of brain tumors Neurobiol Dis 2004 15 437 448 10.1016/j.nbd.2003.11.023 15056451

[B4] Taira AI V Herfkens RJ Gambhir SS Quon A Detection of bone metastases: assessment of integrated FDG PET/CT imaging Radiology 2007 243 204 211 10.1148/radiol.2431052104 17392254

[B5] Even-Sapir E Metser U Mishani E Lievhitz G Lerman H Leibovitch I The detection of bone metastases in patients with high-risk prostate cancer: 99 m Tc-MDP planar bone scintigraphy, single- and multi-field-of-view SPECT, 18F-fluoride PET, and 18F-fluoride PET/CT J Nucl Med 2006 47 287 297 16455635

[B6] Muresan MM Oliver P Leclere J Sirveaux F Brunaud L Klein M Zarnegar R Weryha G Bone metastases from differentitated thyroid carcinoma Endocr Relat Cancer 2008 15 37 49 10.1677/ERC-07-0229 18310274

[B7] Al-Nahhas A Khan S Gogbashian A Banti E Rampin L Rubello D Review. 18F-FDG PET in the diagnosis and follow-up of thyroid malignancy In Vivo 2008 22 109 114 18396792

[B8] Tharp K Israel O Hausmann J Bettman L Martin WH Daitzchman M Sandler MP Delbeke D Impact of 131I-SPECT/CT images obtained with an integrated system in the follow-up of patients with thyroid carcinoma Eur J Nucl Med Mol Imaging 2004 31 1435 1442 15221294 10.1007/s00259-004-1565-2

[B9] Sandu N Schaller B Arasho B Orabi M Wallis interspinous implantation to treat degenerative spinal disease: description of the method and case series Exp Rev Neurother 2011 11 799 807 10.1586/ern.10.187 21651328

[B10] Chin BB Patel P Cohade C Ewertz M Wahl R Ladenson P Recombinant human thyrotropin stimulation of fluoro-D-glucose positron emission tomography uptake in well-differentiated thyroid carcinoma J Clin Endocrinol Metab 2004 89 91 95 10.1210/jc.2003-031027 14715833

[B11] Fili S Karalaki M Schaller B Mechanism of bone metastasis: the role of osteoprotegerin and of the host-tissue microenvironment-related survival factors Cancer Lett 2009 283 10 19 10.1016/j.canlet.2009.01.011 19201081

